# Characteristics of a Hybrid Detector Combined with a Perovskite Active Layer for Indirect X-ray Detection

**DOI:** 10.3390/s20236872

**Published:** 2020-12-01

**Authors:** Hailiang Liu, Jehoon Lee, Jungwon Kang

**Affiliations:** Department of Electronic and Electrical Engineering, Dankook University, Gyeonggi-do 16890, Korea; liuhailiang107@gmail.com (H.L.); usyj0512@gmail.com (J.L.)

**Keywords:** perovskite active layer, indirect X-ray detector, hybrid detector

## Abstract

In this study, we investigated the characteristics of an organic-inorganic hybrid indirect-type X-ray detector with a CH_3_NH_3_PbI_3_ (MAPbI_3_) perovskite active layer. A layer with a thickness of 192 nm annealed at 100 °C showed higher absorption, higher crystallinity, and lower surface roughness than did perovskite layers made under different conditions. In the indirect X-ray detector, a scintillator coupled with the detector to convert X-ray photons to visible photons, and the converted photons were absorbed by the active layer to generate charge carriers. The detector with the optimized MAPbI_3_ (192 nm thick and 100 °C annealing condition) active layer was coupled with a CsI(Tl) scintillator which consisted of 400 μm thick CsI and 0.5 mm thick Al, and achieved the highest sensitivity, i.e., 2.84 mA/Gy·cm^2^. In addition, the highest short-circuit current density (J_SC_), i.e., 18.78 mA/cm^2^, and the highest mobility, i.e., 2.83 × 10^−4^ cm^2^/V·s, were obtained from the same detector without the CsI(Tl) scintillator.

## 1. Introduction

Metal-halide perovskites are promising materials for optoelectronic and photonic devices, such as solar cells [1,2], photodetectors [3,4], and light-emitting diodes [5,6], and their applications are expanding to field-effect transistors [7,8]. These materials have many interesting properties, such as low-cost solution processability [9], highly efficient light absorption [10], long electron-hole diffusion length [11], and high carrier mobility [12]. Among many applications, perovskite solar cells have shown impressive results, with efficiencies of over 20% after just a few years of tremendous effort [13,14]. Methylammonium lead iodide (MAPbI_3_) is not only the most basic material but also the most widely used perovskite material as an active layer for various devices. This material can also be applied to the active layer of X-ray radiation detectors in fields such as security and health care [15,16,17,18]. Recently, Yakunin et al. reported a MAPbI_3_-based p-i-n photodiode with a sensitivity of 0.03 mA/Gy·cm^2^ [19]. Shrestha et al. observed excellent performance for a direct-conversion X-ray detector based on millimeter-thick MAPbI_3_ with a sensitivity of 0.25 mA/Gy·cm^2^ [20]. If the detection sensitivity and manufacturing process are improved, MAPbI_3_ could be a promising candidate for an active layer applied to X-ray detectors.

Recent studies have emphasized the importance of the perovskite morphology [21,22] and crystal orientation [23,24] for the performance of perovskite-applied devices. The smoother surface, high crystallinity, and preferred crystal orientation of the perovskite film generally lead to high photocurrents. In addition, the photovoltaic absorber MAPbI_3_ shows the CH_3_NH_3_ molecular motion, which can induce a dynamic bandgap, could prevent carrier recombination, and could help excitons to separate, thereby improving their efficiencies in optoelectronic devices [25]. A slightly larger grain size and higher crystallinity of MAPbI_3_ helped reduce the trap density and increase the photocurrent of devices. Moreover, solvent annealing helps to obtain a larger MAPbI_3_ grain size and crystallinity, which could lead to significant improvements in material electronic properties and photovoltaic device performance [26]. Adjusting the thickness of the perovskite layer was critical to determine the influence of its morphology [27]. Although the efficiencies of perovskite materials in optoelectronic devices have been extensively improved, no systematic research has been undertaken to determine the influence of the annealing temperature and thickness of the perovskite layer on its surface morphology and sensitivity, especially concerning the performance of X-ray devices.

In this work, we reported a highly sensitive X-ray detector with a thin perovskite active layer for indirect X-ray detection under 63 keV X-ray irradiation conditions. The effects of the annealing temperature and film thickness of the perovskite film on the morphology, light absorption and crystal orientation of the film were investigated, and finally, the influence of these parameters on the operating characteristics of a detector with a perovskite layer was investigated. We analyzed the crystal orientation of the perovskite film using the X-ray diffraction method (XRD, Rigaku D/Max-2500) and light absorbance using UV-Visible absorption spectroscopy (UV-VIS, Optizen 2120UV). The thickness and surface roughness of the perovskite film were measured using scanning electron microscopy (SEM, Hitachi S-4700) and atomic-force microscopy (AFM, Park Systems XE-150). When the perovskite detector was decoupled with a CsI(Tl) scintillator, it worked like a photodetector or solar cell, and the series resistance (R_S_) and short-circuit current (J_SC_) were extracted through the current density-voltage (J-V) characteristics. When the perovskite detector was coupled with the CsI(Tl) scintillator, it worked like an X-ray detector, and the X-ray parameters were extracted under X-ray irradiation. The CsI(Tl) scintillator consisting of 400 μm thick CsI and 0.5 mm thick Al showed a maximum emission peak at 550 nm, matching with the absorption spectrum of the MAPbI_3_ active layer (as shown in Figure S1 in Supplementary Materials). The CsI(Tl) scintillator served to convert incident X-ray photons into visible photons, and the converted visible light photons were absorbed by the perovskite active layer to create charge carriers.

**Figure 1:**
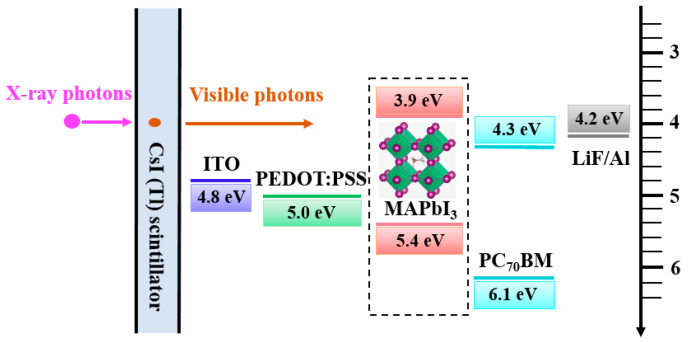
Energy-band diagram of the proposed detector with perovskite active layer.

Figure 1 shows the energy-band diagram of the proposed detector with the MAPbI_3_ active layer. The visible photons converted by the scintillator were absorbed by the MAPbI_3_ to create electron-hole pairs that were separated in the active layer according to the energy band position. The electrons were transferred to the indium-tin oxide (ITO) anode, where the resulting in charge was collected. As the electron transport layer (ETL), (6,6)-phenyl-C71-butyric acid methyl ester (PCBM) was used, while poly(3,4-ethylenedioxythiophene) polystyrene sulfonate (PEDOT:PSS) was used as the hole transport layer (HTL), which helped the movement of the electrons and holes to the electrodes, respectively.

## 2. Experimental Preparations

### 2.1. Synthesis of MAPbI_3_ Precursor Solution

**Figure 2:**
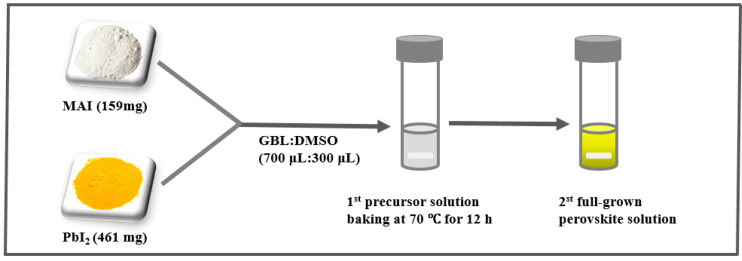
Solvent engineering procedure for the preparation of the perovskite precursor solution.

The compounds iodide (MAI, Aldrich 793493), lead (II) iodide (PbI_2_, Aldrich 203602), γ-butyrolactone (GBL, Aldrich H7629), dimethyl sulfoxide (DMSO, Aldrich D8418) and chlorobenzene (Anhydrous, Aldrich 284513) were used as received. We dissolved MAI (159 mg) and PbI_2_ (461 mg) at 1:1 (mol/mol) in GBL (700 μL) and DMSO (300 μL) at 7:3 (vol/vol) and stirred it at 70 °C for 12 h. Finally, we got the full-grown mature precursor solution. The complete solvent engineering procedure for the perovskite precursor solution is shown in Figure 2. The perovskite precursor solution was prepared referring to the method reported in [28].

### 2.2. Devices Fabrication

**Figure 3:**
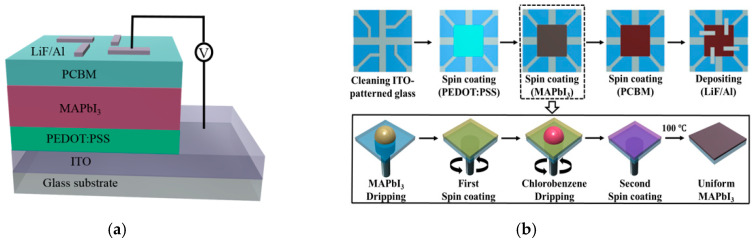
(**a**) Schematics of the constructed bulk heterojunction preoskite device, (**b**) Fabrication procedure of the perovskite detector and solvent engineering procedure for preparing the uniform and dense perovskite film.

Figure 3a shows a schematic diagram of the fabricated detector, which consisted of glass/ITO/PEDOT:PSS/MAPbI_3_ perovskite active layer/PCBM/LiF/Al. Figure 3b shows the fabrication procedure of the proposed detector with the perovskite active layer and the solution process to form the layer. An indium-tin-oxide (ITO) anode (150 nm) on a glass substrate was patterned and then cleaned sequentially for 5 min using sonification treatment of acetone, methanol and IPA. The glass substrate was dried in a vacuum oven at 100 °C for 10 min. A charge transport layer of poly(3,4-ethylene dioxythiophene):poly (styrene sulfonate) (PEDOT:PSS, Clevios P VP Al4083) layer was spin-coated on the cleaned glass substrate at 3000 rpm for 30 s, and then annealed at 150 °C for 30 min. The thickness of the PEDOT:PSS layer as the hole transport layer (HTL) was about 30 nm. The prepared MAPbI_3_ solution was spin-coated on the PEDOT:PSS layer at various spin-rates of 3000, 3500, 4000, and 4500 rpm for 40 s. Before the end of the spin-coating process, 200 μL chlorobenzene solvent was pipetted onto the substrate to wash off the DMSO solvent of the perovskite wet film and induce rapid crystallization. After the spin-coating process, the perovskite film was baked at 100 °C for 10 min. Then, 20 mg of phenyl-C71-butyric acid methyl ester (PCBM, Emindex 609771-63-3) powder was dissolved in 1 mL of chlorobenzene and stirred at 60 °C for 3 h. The PCBM solution was spin-coated onto the perovskite layer at a spin rate of 1100 rpm, and the perovskite was baked at 100 °C for 10 min. The thickness of the PCBM layer as the electron transport layer (ETL) was about 80 nm. After forming the PCBM layer, the aging time of the sample was 12 h. LiF/Al as the cathode, consisting of 5 nm thick LiF and 120 nm thick Al, was deposited on the PCBM layer in a thermal evaporation chamber. The manufactured detector was encapsulated with a glass cover to prevent it from being exposed to air. Finally, the detector with four effective areas of 0.04 mm^2^ was successfully fabricated.

### 2.3. Experimental Set-Up

**Figure 4:**
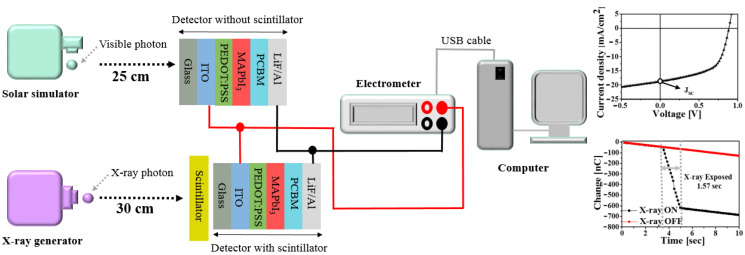
Schematic diagram of the experimental setup for measuring the parameters of the perovskite detector without and with the CsI(Tl) scintillator.

Figure 4 shows the experimental setup to evaluate the properties of the radiation detector with the perovskite active layer. It mainly consisted of three components: an X-ray generator (AJEX 2000H), a solar illumination simulator (San Ei Elec. XES-40S2-CE), and an electrometer (Keithley 2400) to measure the photocurrent.

First, the characteristics of the detector without the CsI(Tl) scintillator were evaluated under the condition of exposure to the AM 1.5G filtered Xe lamp of the solar simulator, and the intensity of the exposed light was 100 mW/cm^2^. The distance between the solar simulator and the detector was fixed at 25 cm. The generated charges were collected by applying a bias of −1.0 to 1.0 V. The various parameters, such as short-circuited current density (J_SC_) and series resistance (R_S_), were obtained from the J-V characteristics during the artificial solar irradiation. J_SC_ is the current density as measured by the incident light when the detector was short-circuited, which could be extracted from the 0 V bias of the J-V curve. R_S_ is the resistance of the active layer outside its space-charge layer, in addition to the contact resistances of the metal electrodes to the active layer. They are crucial for the charge-carrier generation of the detector.

Second, for the X-ray detector measurement, we evaluated the perovskite detector combined with the CsI(Tl) scintillator under X-ray exposure. For all experiments, the operation of the X-ray generator was fixed at 80 kVp and 60 mAs, and it was irradiated for 1.57 s. The distance between the X-ray generator and the detector was 30 cm. The exposure X-ray dose was measured using an ion chamber (Capintec CII50) at the same distance. The absorbed dose was converted from X-ray exposure, which was 3.44 mGy. To collect the charge generated during X-ray exposure, a 0.6 V bias was applied between the cathode and anode of the detector. We calculated the radiation parameters during the X-ray exposure, that is, the collected current density (CCD) during the X-ray irradiation on-condition and the dark current density (DCD) during the X-ray irradiation off-condition were calculated by Equations (1), and (2), respectively. The sensitivity was calculated using Equation (3), which represented the generated current in proportion to the absorbed dose.

(1) CCD [μAcm2]= Collected Current during X−ray ONExposed Detection Area

(2)  DCD [μAcm2]= Collected Current during X−ray OFFExposed Detection Area

(3)  Sensitivity [μAmGy·cm2]= CCD−DCDAbsorbed Dose

## 3. Results and Discussion

**Figure 5:**
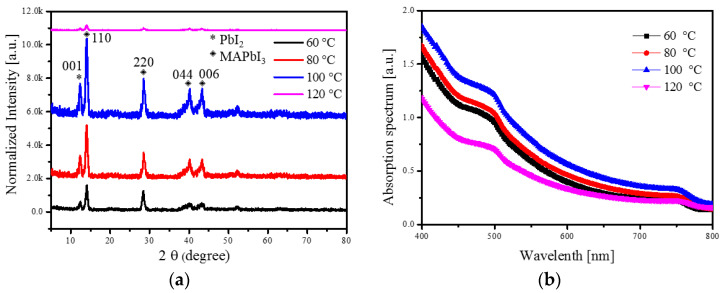
(**a**) XRD patterns and (**b**) absorption spectra of 192 nm thick MAPbI_3_ layer with different annealing temperatures.

We used X-ray diffraction (XRD) measurements to investigate the properties of the 192 nm thick MAPbI_3_ layer under different annealing temperatures (60, 80, 100 and 120 °C), as shown in Figure 5a. Noticeable diffraction peaks were observed depending on the annealing temperature. The XRD patterns were composed of two phases, i.e., MAPbI_3_ and PbI_2_. For the PbI_2_ phase, we assigned the peak at 12.21° to the (001) plane. For the MAPbI_3_ phase, the peaks at 14.08, 28.46, 40.21 and 43.31° were respectively assigned to the (110), (220), (044) and (006) planes. The highest peak intensity of the perovskite layer was achieved at 100 °C. As the annealing temperature of the perovskite increased, the rate of perovskite crystallization was similarly increased, and the crystallinity improved up to the optimal temperature of 100 °C. However, when the annealing temperature exceeded the optimal temperature, i.e., up to 120 °C, the crystallinity dropped significantly, because the MAPbI_3_ was decomposed into PbI_2_, CH_3_NH_2_ and HI at an annealing temperature of 120 °C [26].

Figure 5b shows the absorption spectra of the 192 nm thick MAPbI_3_ layer with annealing temperatures of 60, 80, 100, and 120 °C. It was clear that the absorption intensity increased as the annealing temperature increased from 60 to 100 °C. When the annealing temperature reached 120 °C, the annealing temperature was too high, resulting in degradation in the perovskite layer. As a result, the absorption intensity decreased as the light-harvesting ability decreased. The result of the decrease of light absorption capacity was consistent with the result of XRD, which was caused by the decomposition of the MAPbI_3_ due to the high heating temperature.

To investigate the photovoltaic properties of the detectors based on a MAPbI_3_ layer under different annealing temperatures, we measured the current density-voltage (J-V) characteristics of the detector based on a 192 nm thick MAPbI_3_ layer, as shown in the Figure S4. The parameters including J_SC_, R_S_, CCD and sensitivity of the detectors are listed in Table 1. The device constructed with an annealing temperature of 60 °C achieved J_SC_ of 16.39 mAcm^−2^, R_S_ of 212.78 Ω, and sensitivity of 2.26 mA/Gycm^2^. When the active layer was annealed at 100 °C, the device yielded an improved J_SC_ of 18.78 mAcm^−2^, R_S_ of 172.23 Ω, and sensitivity of 2.84 mA/Gycm^2^, i.e., 25% better sensitivity compared to the use of an active layer annealed at 60 °C. In addition, to further observe the effect of different temperatures on the perovskite layer, the current density-voltage (J-V) curves of the detectors with different thicknesses of MAPbI_3_ active layer (109, 145 and 215 nm) were also measured (as depicted in Figures S2, S3 and S5 of Supplementary Materials). The parameters such as J_SC_, R_S_, CCD and sensitivity of the detectors are listed in Table S1–S3 (see Supplementary Materials), respectively. About 10 detectors with the MAPbI_3_ active layer were manufactured to verify the reproducibility.

**Figure 6:**
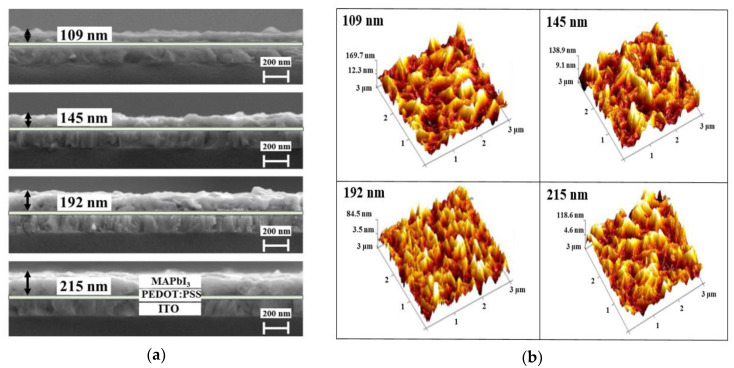
(**a**) Cross-sectional SEM images and (**b**) AFM images of MAPbI_3_ layer with different thicknesses at an annealing temperature 100 °C.

Scanning electron microscopy (SEM) images of MAPbI_3_ films of different thicknesses grown on a glass/ITO/PEDOT: PSS stacked structure are shown in Figure 6a. The annealing temperature was fixed at 100 °C. The MAPbI_3_ film thickness depended on the spin-coating speed. From the cross-sectional SEM images, we confirmed that MAPbI_3_ films that were 109, 145, 192, or 215 nm thick were sequentially formed under spin rates of 3000, 3500, 4000, and 4500 rpm. To understand the effect of perovskite film thickness on the morphology, we used AFM analysis to evaluate the change in the topology of the perovskite films on the ITO-coated glass. Figure 6b shows the topographical 3D images of the perovskite thin films. The RMS surface roughness (Rq) of 109 nm, 145 nm, 192 nm, and 215 nm thick perovskite layers was 43.8 nm, 32.2 nm, 21.9 nm, and 36.8 nm, respectively. As the thickness of the perovskite film increased, the surface roughness decreased. This was because the presolution of MAPbI_3_ could grow more completely into a thin film. When the thickness increased to 215 nm, the surface roughness increased; this was because some of the presolution could not react completely with an excessively thick MAPbI_3_ layer.

**Figure 7:**
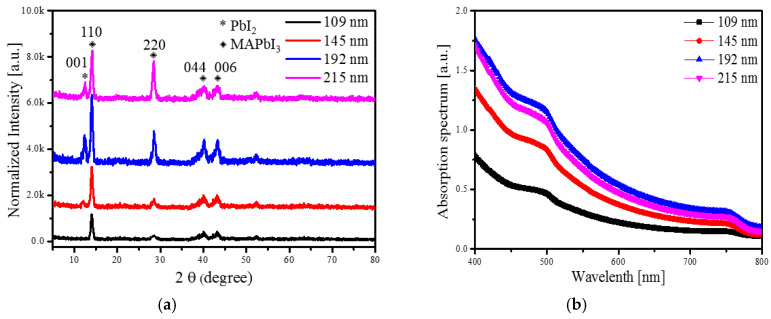
(**a**) XRD patterns and (**b**) absorption spectra of MAPbI_3_ layer with different thicknesses at an annealing temperature 100 °C.

Figure 7a shows the XRD characteristics of MAPbI_3_ films with different thicknesses at an annealing temperature of 100 °C. For all MAPbI_3_ peaks, the (110) phase had the highest peak intensity, while the other (220), (044) and (006) phases had relatively low peak intensities. It was confirmed that the polycrystalline MAPbI_3_ film had a dominant orientation in the (110) phase. As the thickness of the MAPbI_3_ film increased, the peak intensity of the (110) phase increased, and it was confirmed that the change in the film thickness influenced the crystallinity of the perovskite. Considering the structural stability for the (110) orientation, the 192-nm thick MAPbI_3_ film had higher structural stability than did the films with other thicknesses. The preferred (110) orientation of the MAPbI_3_ layer had superior photoelectric properties because of the improved crystalline quality.

Good absorption properties in the visible region of the active layer are important. To further understand the light absorption capacity of perovskite layers, we measured the absorption spectra of MAPbI_3_ layers with different thicknesses annealed at 100 °C. Figure 7b shows the absorbance curves of the perovskite layers. The absorbance of the perovskite film increased with increasing thickness. The highest absorbance was obtained with a 192 nm thick perovskite layer. When the thickness of the perovskite film exceeded 192 nm, the previous AFM and XRD measurements showed an increase in surface roughness and a decrease in crystallinity.

**Figure 8:**
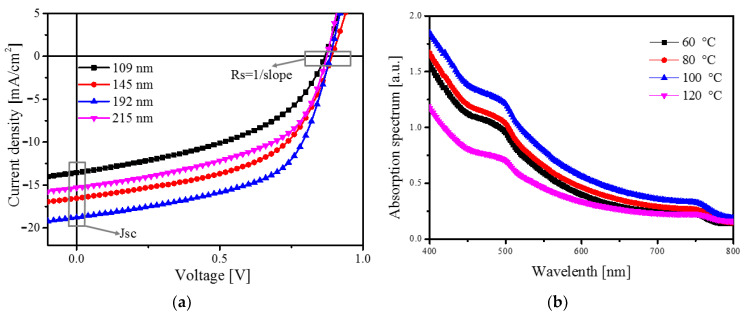
(**a**) Current density-voltage (J-V) characteristics of the detectors based on a MAPbI_3_ layer annealed at temperature 100 °C, and (**b**) curve fitting of the logarithmic J-V characteristics of the detectors in the dark.

To investigate the photovoltaic properties of the detectors with different thicknesses of the MAPbI_3_ layer, we measured the current density-voltage (J-V) characteristics using the solar simulator and electrometer mentioned in Section 2.3. The J-V curves of the detectors with different thicknesses of perovskite layers are shown in Figure 8a, and parameters such as Jsc and Rs extracted from the J-V curves are listed in Table 2. The maximum J_SC_ of 18.78 mA/cm^2^ and the minimum series resistance of 172.23 Ω were obtained with a film thickness of 192 nm. The optimum thickness of the active layer to improve the detector performance was selected in consideration of the following three phenomena: light-absorption [29], carrier-transport, and carrier-loss characteristics [30]. For example, a mismatch between the carrier diffusion-length and the active layer thickness can lead to a decrease in the current density (J) through carrier-loss.

Figure 8b shows the logarithmic J-V characteristics of the detectors with different thicknesses of the perovskite layers. The effect of perovskite thickness on detector performance was studied by analyzing carrier mobility. The carrier mobility was determined using the space-charge-limited-current (SCLC) method in the dark [31], and using the modified Mott-Gurney equation, as shown below:

(4) $$\mu =\frac{8}{9}\cdot J\cdot \frac{L^{3}}{V_{a}{}^{2}\cdot \varepsilon_{0}\cdot \varepsilon_{r}}$$

where *ε*_0_ is the permittivity of free space, *ε_r_* is the relative permittivity of the perovskite layer, *V_a_* is the voltage applied across the detector, *μ* is the carrier mobility, and *L* is the thickness of the perovskite layer. The calculated mobility listed in Table 2 was 1.21 × 10^−4^, 1.97 × 10^−4^, 2.83 × 10^−4^, and 1.83 × 10^−4^ cm^2^/Vs for the detectors with perovskite layers with thicknesses of 109, 145, 192 and 215 nm, respectively. For example, Li. et al. successfully prepared a solar cell with a MAPbI_3_ active layer, and obtained SCLC mobility of 1.90 × 10^−4^ cm^2^/Vs [32]. As the thickness of the perovskite film increased, the carrier mobility increased. When the thickness of the perovskite film exceeded the optimal 192 nm thickness, the surface roughness increased and the peak of the (110) plane decreased. The mobility along the (100) direction was larger than the mobility along (001) or other directions [33], which contributed to the formation of a short migration path and increased charge extraction and collection.

**Figure 9:**
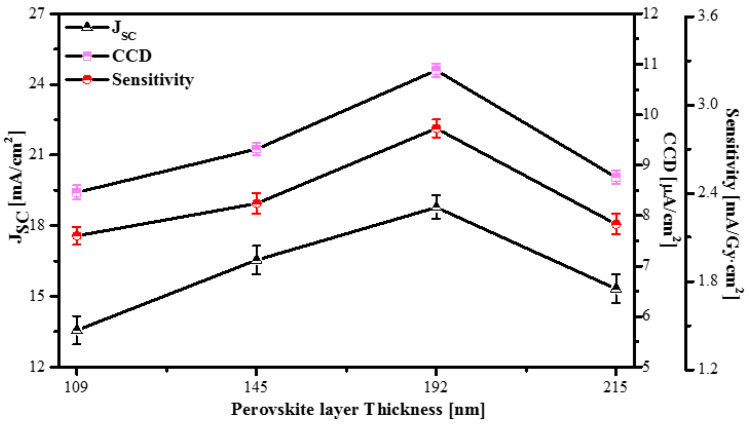
J_SC_, CCD, and sensitivity of the detector based on MAPbI_3_ layer with different thicknesses at an annealing temperature 100 °C. Error bars represent the standard deviation based on 10 devices per condition.

We used the X-ray generator and electrometer mentioned in Section 2.3 to study the radiation properties of the detectors with different thicknesses of the MAPbI_3_ layer. With the scintillator-decoupled detector under artificial solar exposure, the J_SC_ was obtained via the 0 V bias of the J-V curves. We found the J_SC_ to be 13.56, 16.54, 18.78 and 15.32 mA/cm^2^ for the X-ray detectors with perovskite layers with thicknesses of 109, 145, 192, and 215 nm, respectively, as indicated in Figure 9 (left axis). We calculated the CCD and sensitivity using Equations (1) and (2) in Section 2.3. The X-ray detectors with 109, 145, 192, and 215 nm thick perovskite layers exhibited sensitivities of 2.11, 2.33, 2.84, and 2.19 mA/Gycm^2^, respectively (right axis in Figure 9). The highest sensitivity, i.e., 2.84 mA/Gycm^2^, under X-ray exposure was achieved with the detector with a 192 nm thick perovskite layer. Starkenburg et al. successfully prepared an X-ray detector based on a boron subphthalcyanine chloride (SubPc):PC60BM active layer combined with a CsI scintillator, and obtained a sensitivity of 0.96 mA/Gycm^2^ at a bias voltage of 1 V [34]. High sensitivity was obtained from the detector by applying the optimal conditions of annealing temperature and thickness, thereby improving the surface roughness, increasing the light absorption capacity, improving mobility, and improving the recombination loss of the perovskite layer.

## 4. Conclusions

In this paper, we investigated a high-sensitivity X-ray detector with a perovskite active layer for indirect X-ray detection. By changing the annealing temperature and film thickness, we could optimize the electronic and optical properties of the perovskite film applied to the active layer of the detector. First, we studied the structural properties of the MAPbI_3_ films prepared with different annealing temperatures (60, 80, 100, and 120 °C). The MAPbI_3_ film showed the highest crystallinity and absorption intensity with an annealing temperature of 100 °C. The detector with the 100 °C annealed perovskite film as the active layer had better photoelectric properties than the detector with the films annealed at other temperatures. Second, by studying the characteristics of different thicknesses (109, 145, 192 and 215 nm) of the perovskite layer, we found that the 192 nm thick layer had lowest roughness and better mobility. The detector with the perovskite layer that was 192 nm thick had better current density because of its high light-harvesting efficiency, long electron-hole diffusion length, and less carrier-recombination loss. Finally, the highest sensitivity, i.e., 2.84 mA/Gycm^2^, was obtained using the detector with the optimized perovskite layer. Given the many interesting properties of perovskite, such as its low-cost solution processability, highly efficient light absorption, long electron−hole diffusion length, and high carrier mobility, MAPbI_3_ is a promising candidate for use as an active layer applied to X-ray detectors.

## Figures and Tables

**Table 1:** J_SC_, R_S_, CCD and sensitivity of the detector based on 192 nm thick MAPbI_3_ layer at various annealing temperatures.

Temperature [°C]	Jsc [mA/cm^2^]	Rs [Ω]	CCD [μA/cm^2^]	Sensitivity [mA/Gy·cm^2^]
60	16.39 ± 0.65	212.78 ± 3.9	8.78 + 0.14	2.26 ± 0.07
80	17.74 ± 0.62	181.72 ± 3.8	9.41 + 0.13	2.43 ± 0.06
100	18.78 ± 0.58	172.23 ± 3.9	10.88 + 0.13	2.84 ± 0.06
120	11.56 ± 0.72	298.87 ± 3.8	7.82 + 0.13	1.99 ± 0.08

**Table 2:** J_SC_, R_S_ and mobility of the detector based on MAPbI_3_ layer with different thicknesses at an annealing temperature 100 °C.

Layer Thickness [nm].	J_SC_ [mA/cm^2^]	Rs [Ω]	Mobility [cm^2^/V·s]
109	13.56 ± 0.63	282.11 ± 3.9	(1.21 ± 0.06) × 10^−4^
145	16.54 ± 0.61	198.36 ± 3.8	(1.97 ± 0.04) × 10^−4^
192	18.78 ± 0.58	172.23 ± 3.9	(2.84 ± 0.04) × 10^−4^
215	15.32 ± 0.62	256.09 ± 3.8	(1.83 ± 0.05) × 10^−4^

## Supplementary Materials

The following are available online at https://www.mdpi.com/1424-8220/20/23/6872/s1.
