# Supplementary material for: Characteristics of a Hybrid Detector Combined with a Perovskite Active Layer for Indirect X-ray Detection
Source: Sensors (Basel). 2020 Dec 1;20(23):6872. doi: 10.3390/s20236872 (PMC7730663; doi:10.3390/s20236872)
Supplement: Supplementary file 1 [file sensors-20-06872-s001.pdf]

SUPPLEMENTARY INFORMATION
Characteristics of a hybrid detector combined with a perovskite active layer for indirect X-ray detection

Hailiang Liu, Jehoon Lee and Jungwon Kang*

Department of Electronic and Electrical Engineering, Dankook University, Gyeonggi-do 16890, Korea; liuhailiang107@gmail.com (H.L.); usyj0512@gmail.com (J.L.); jkang@dankook.ac.kr (J.K.)

* Correspondence: jkang@dankook.ac.kr; Tel.: +82-31-8005-3624 (J.K)

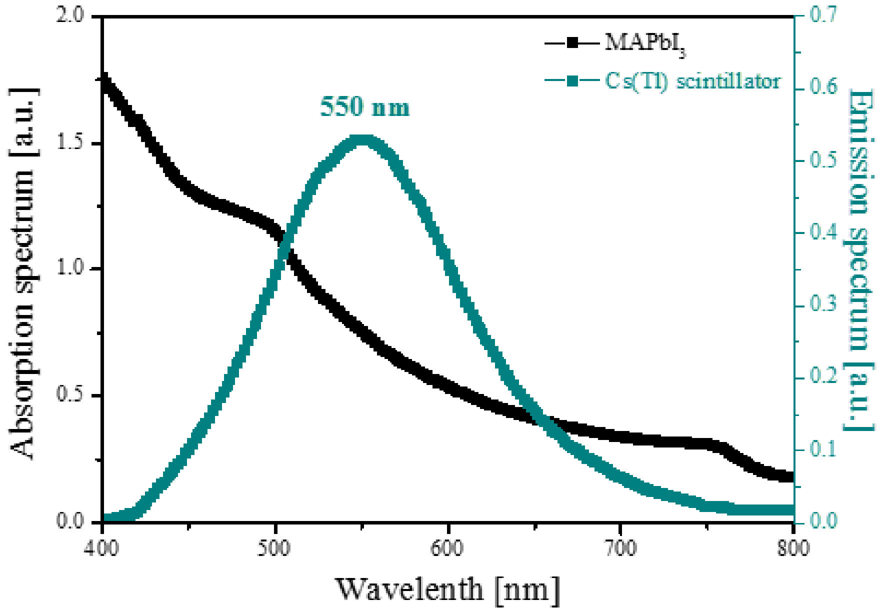

Figure S1. Absorption spectrum of the optimized perovskite layer with a thickness of 192 nm annealed at 100℃ and the emission spectrum of the CsI(Tl) scintillator.

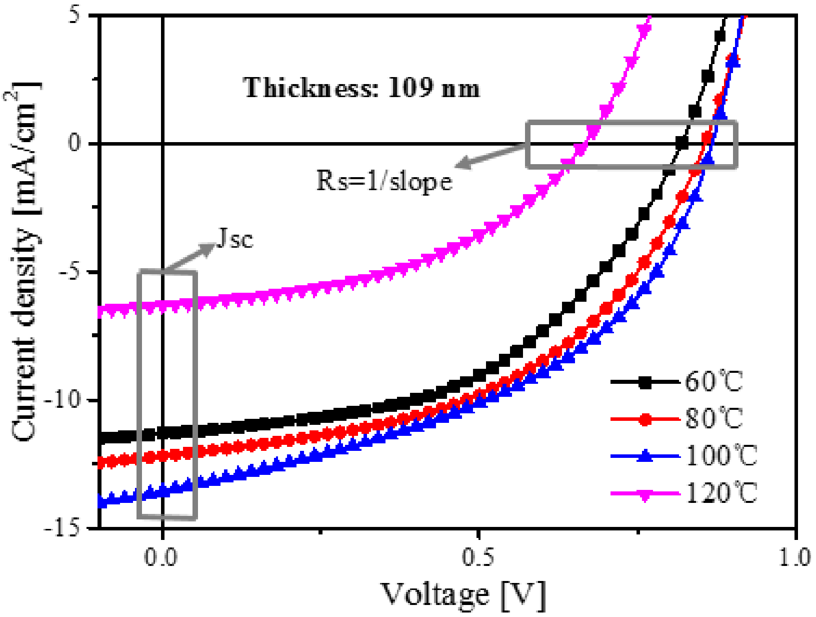

Figure S2. Current density-voltage (J-V) characteristics of the detectors based on 109-nm thick $MAPbI_3$ under different annealing temperatures.

Table S1. J_{SC}, R_S, CCD and Sensitivity of the detectors based on 109-nm thick $MAPbI_3$ under different annealing temperatures.

Temperature [℃]	J_{SC} [mA/cm^2]	R_S [Ω]	CCD [μA/cm^2]	Sensitivity [mA/Gy·cm^2]
60	11.31 ±0.67	301.13 ±3.7	6.62 ±0.12	1.68 ±0.05
80	12.18 ±0.65	292.19 ±3.7	7.79 ±0.13	2.01 ±0.06
100	13.56 ±0.63	282.11 ±3.9	8.46 ±0.14	2.11 ±0.06
120	6.32 ±0.71	381.23 ±4.0	5.47 ±0.14	1.35 ±0.07

The difference value between positive and negative in all tables represent the standard deviation based on 10 devices per condition.

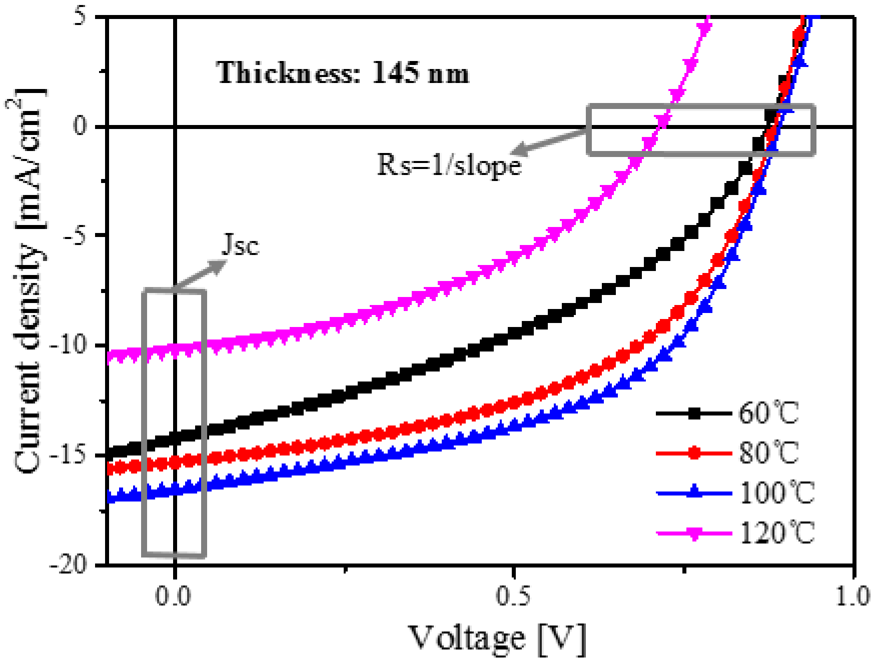

Figure S3. Current density-voltage (J-V) characteristics of the detectors based on 145-nm thick $MAPbI_3$ under different annealing temperatures.

Table S2. J_{SC}, R_S, CCD and Sensitivity of the detectors based on 145-nm thick $MAPbI_3$ under different annealing temperatures.

Temperature [℃]	J_{SC} [mA/cm^2]	R_S [Ω]	CCD [μA/cm^2]	Sensitivity [mA/Gy·cm^2]
60	14.23 ± 0.64	261.73 ± 3.8	8.32 ± 0.11	2.14 ± 0.06
80	15.31 ± 0.62	258.13 ± 3.7	8.47 ± 0.12	2.18 ± 0.05
100	16.54 ± 0.61	198.36 ± 3.8	9.32 ± 0.13	2.33 ± 0.07
120	10.17 ± 0.68	316.54 ± 3.9	6.22 ±0.14	1.57 ± 0.08

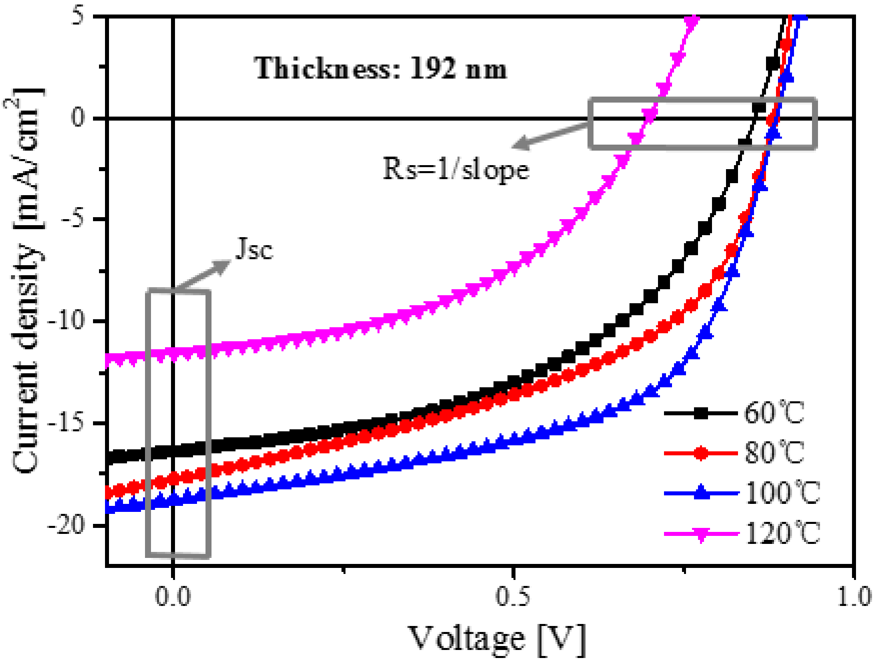

Figure S4. Current density-voltage (J-V) characteristics of the detectors based on 192-nm thick $MAPbI_3$ under different annealing temperatures.

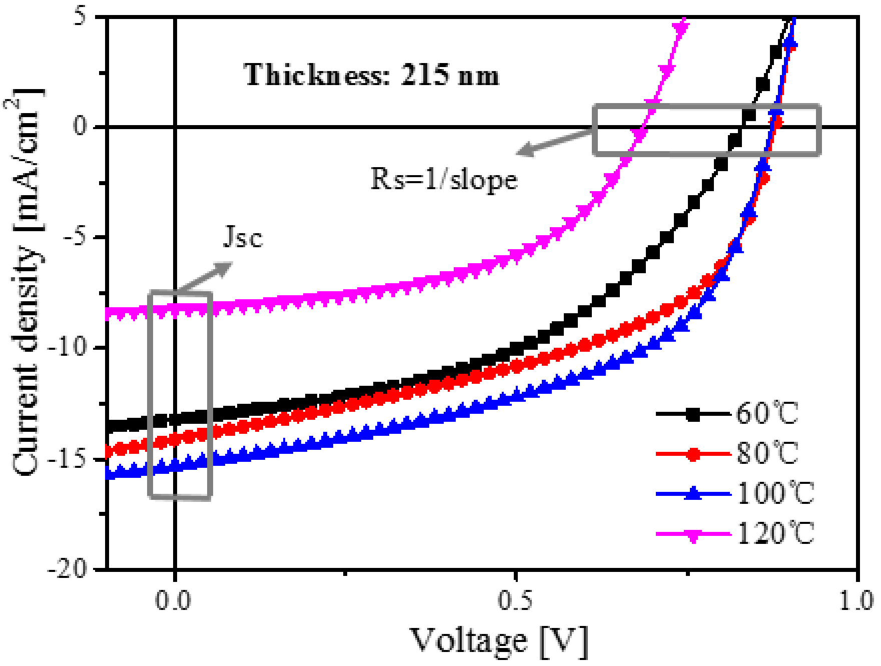

Figure S5. Current density-voltage (J-V) characteristics of the detectors based on 215-nm thick $MAPbI_3$ under different annealing temperatures.

Table S3. J_{SC}, R_S, CCD and Sensitivity of the detectors based on 215-nm thick $MAPbI_3$ under different annealing temperatures.

**Temperature [℃]**	**J_{SC} [mA/cm^2]**	**R_S [Ω]**	**CCD [μA/cm^2]**	**Sensitivity [mA/Gy·cm^2]**
60	13.23 ± 0.66	274.78 ± 3.7	8.06 ± 0.13	2.06 ± 0.08
80	14.13 ± 0.64	268.32 ± 3.8	8.35 ± 0.12	2.13 ± 0.09
100	15.32 ± 0.62	256.09 ± 3.8	8.76 ± 0.13	2.19 ± 0.07
120	8.26 ± 0.69	341.78 ± 3.9	5.93 ± 0.14	1.47 ± 0.10

© 2020 by the authors. Licensee MDPI, Basel, Switzerland. This article is an open access article distributed under the terms and conditions of the Creative Commons Attribution (CC BY) license (http://creativecommons.org/licenses/by/4.0/).
